# Report of Operation for Hæmatoma

**Published:** 1889-01

**Authors:** J. McFadden Gaston

**Affiliations:** At the Clinic of the Southern Medical College


### REPORT OF OPERATION FOR HÆMATOMA.

BY PROFESSOR J. MCFADDEN GASTON, M. D.

At the Clinic of the Southern Medical College on Wednesday, 21st of November.

The case of Cornelius White, aged 50 years was presented, being hæmatoma of nine weeks standing, resulting from a pistol shot wound of the thigh. The ball entered about the junction of the lower and middle third of the anterior aspect, ranging obliquely upwards across the inner portion, and had been cut out beneath the skin shortly after the accident. Some blood had been discharged from both wounds from time to time and with distension of the deep-seated and superficial structures led to the conclusion that there was a considerable collection of blood in the cellular tissue. The general condition of the patient indicated that a serious result must ensue without an operation, and that perhaps septic contamination had already began, which would preclude any salutary outcome from the removal of the blood clot, but as this was the only hope for saving the life of the patient, he had consented to have the operation performed.

Professor J. McFadden Gaston laid open the integument to an extent of eight inches in a line upward from the point of entrance of the ball, and scooped out with his hands an immense accumulation of clotted blood, while the femoral artery was controlled by a tourniquet below the pubic bone. The main artery was found to be open to an extent of half an inch, and the blood escaped from the cardiac and distal portions, so that it became necessary to ligate it above and below the injury. A strong iron-dyed silk ligature No. 13 was tied three quarters of an inch above, and another at a similar distance below the opening, but owing to the dense condition of the walls of the femoral did not close entirely its lumen, and it became necessary to apply two others having several strands of silk with all the force that could be used for the complete arrest of flow of blood.

The extensive cavity was then thoroughly washed out with a warm solution of chloride of sodium forced into all the crevices of the distended cellular tissues, followed by an injection of a carbolic solution. A piece of antiseptic gauze was then saturated with a solution of spts turpentine f ℥ i, and camphor f ʒ i, and pressed within the cavity so as to come in contact with every part, swabbing it out completely; this being removed a fresh piece soaked with this antiseptic was left within and two drainage tubes were lodged in the cavity so as to allow a discharge from the upper and lower extremity of the incision. A single stitch was taken through the integument near each tube so as to retain them in position, leaving the intervening portions of the incision without stitches to admit of free exudation from the deep-seated parts. A dressing with iodoform and antiseptic gauze was covered with absorbent cotton, secured by a roller bandage. The entire lower extremity was also enveloped with cotton and supported by a roller, with directions to keep hot brick to the foot and leg. Dr. Gaston was assisted in the various steps of this operation by Drs. Nicolson, Elkin, W. D. and B. W. Bizzell, Murray, Stockton and Joye. √

On November 22nd, the dressings were found saturated with bloody serum, which had escaped from the line of the incision and from the drainage tubes. The gauze which had been saturated with the camphorated turpentine and left in the cavity was removed, and a thorough washing out with a strong solution of chloride of sodium thrown in by a syringe was followed by an injection of carbolized water. A layer of antiseptic gauze covered with iodoform was placed on the line of incision, and the thigh was enveloped with cotton, secured by a roller bandage.

It was observed that there was very little if any diminution of the temperature of the limb, and the general œdema has lessened so much as to require an application of the roller from the foot up to the dressing of the thigh.

On November 29th. The discharge had diminished so much as to dispose with one of the drainage tubes, and the general condition of the patient gave promise of a favorable result, thus affording encouragement to evacuating collections of blood from injury to the femoral and ligation of the artery for the prevention of septicæmia, even after lapse of two months from the infliction of the wound. Thc injection of a solution of peroxide of hydrogen was now used in place of the carbolized water as a correction of the purulent collection.

December 12th. The suppuration was notably diminished under the influence of peroxide of hydrogen, and was of a more healthy character, but the ligatures had not separated and the patient was again presented to the class for the adoption of measures of relief in this respect. Dr. Gaston, with the assistance of Drs. B. W. Bizzell passed an aneurism needle within the loop of the two ligatures on the cardiac portion of the femoral and divided them with the scissors, thus getting rid of those ligatures. The two ligatures on the distal portion being shut in by adhesions of the skin so as to preclude this mode of proceedure pieces of rubber tubing were attached to them and secured to thigh by adhesive plaster, so as to keep up constant distension. He is ordered mur. tinct. of iron and tinct. of nux vomica.

December 19th. The ligatures have not yet come away but the suppuration is slight throughout the tract of the wound and the favorable condition of the patient under the ferruginous tonic all promise a salutary result of the operation.

December 22nd. The tension upon the ligatures having been kept up for ten days without releasing them from the artery, Dr. Gaston used an ordinary button hook for securing the loops, and then cut them with a narrow bistoury near to the knots so that they were brought away. The length of each loop when detached, measured one inch and a half. The thickening of the coats of the femoral increased the diameter to this extent, even when the lumen was obliterated by the constriction.

There is still a communication from the upper opening of the incision to the point at which these ligatures were removed, from which a small quantity of pus escapes, and this is washed out with a weak carbolic solution. The general condition of the patient is decidedly better, and he has been sitting up out of bed for the past few days.

December 24th. There is a very slight discharge of sero-purulent nature, and the line of incision is closed excepting the openings through which the ligatures were removed. The dressings with carbolic wash, iodoform and cotton are continued for the present, but the case requires no further surgical attention.

In view of the unfavorable conditions surrounding the patient at the time of the operation, and subsequently, this result is highly satisfactory to all concerned.

